# A high burden of adverse life events and poor coping mechanisms experienced by urban-dwelling black South Africans

**DOI:** 10.1371/journal.pone.0238320

**Published:** 2020-09-10

**Authors:** Nasheeta Peer, Carl Lombard, Krisela Steyn, Naomi Levitt

**Affiliations:** 1 Non-communicable Diseases Research Unit, South African Medical Research Council (SAMRC), Durban, South Africa; 2 Department of Medicine, University of Cape Town (UCT), Cape Town, South Africa; 3 Biostatistics Unit, SAMRC, Tygerberg, South Africa; 4 School of Public Health and Family Medicine, UCT, Cape Town, South Africa; 5 Faculty of Medicine and Health Sciences, Division of Epidemiology and Biostatistics, Department of Global Health, Stellenbosch University, Tygerberg, South Africa; 6 Department of Medicine, Chronic Disease Initiative for Africa, UCT, Cape Town, South Africa; Fordham University, UNITED STATES

## Abstract

**Aim:**

In view of the current context of poverty and socio-economic inequalities and the high and rising burdens of HIV infection and non-communicable diseases in South Africa, this study aims to describe the distribution of adverse life events (ALEs) by age and gender, and examine the socio-demographic characteristics, psychosocial coping mechanisms, risky lifestyle behaviours and family burden of HIV-related ill-health associated with ALEs in 25-74-year-old black residents of Cape Town.

**Materials and methods:**

In a random cross-sectional sample, 12 ALEs, tobacco and alcohol use, sense of coherence (SOC), locus of control (LOC) and impact of HIV in the family were determined by administered questionnaires. Data analyses included descriptive statistics adjusted for the realised sample. Multivariable linear regression models assessed the independent associations of increasing number of ALEs.

**Results:**

Among 1099 participants, mean lifetime score of ALE categories examined was 6.1 ±2.1 (range 0–12) with men reporting significantly higher number of events compared with women (p<0.001). The most frequent ALE was the death of a loved one (88.5%) followed by a major financial crisis (81.2%) with no trend across gender or age group. In the multivariable linear regression model, increasing ALEs were significantly associated with male gender, unemployment, having spent >50% of life in urban areas, >7 years of education, problematic alcohol use and poorer psychosocial coping mechanisms defined by low SOC and LOC. All four variables pertaining to HIV-related burden of ill-health in the family were significantly associated with increasing ALEs.

**Conclusions:**

Considering that lower SOC and LOC and problem drinking were significantly linked to ALEs, policymakers need to formulate strategies that improve coping mechanisms and promote problem-solving behaviours, target the high burden of alcohol misuse and address unemployment.

## Introduction

In South Africa, a country emerging from the toxic policies of apartheid that aggressively subjugated most of its population till the introduction of democracy in 1994, the toll of apartheid still resonates today [1]. It struggles with multiple health-related and socioeconomic ills that are dominated by HIV/AIDS, and high levels of poverty, crime, violence and unemployment. This is likely to leave South Africans feeling helpless, unempowered and struggling to cope with their daily lives. These socioeconomic woes, among others, are key contributors to ill-health in the country [2].

Moreover, the unscientific health policies pertaining to the HIV epidemic during Thabo Mbeki’s presidency (1999–2008) did nothing to improve the psyche of South Africans. This era witnessed a failure to provide antiretroviral therapy to those with HIV/AIDS with the subsequent loss of hundreds of thousands of lives [1, 2]. HIV/AIDS was largely responsible for worsening mortality in most age groups between 1990 and 2005 [2]. Despite mortality declining with the introduction of antiretroviral therapy, HIV still accounted for 23.4% of deaths in South Africa in 2019 and almost eight million adults were HIV positive [3].

In addition to the direct burden that HIV inflicts on the infected, it has far-reaching and devastating social, psychological and economic consequences on the families of the afflicted. Children are orphaned when their parents succumb to HIV/AIDS leaving grandparents with the responsibility of their care [4]. This has a profound negative effect on all affected.

Many of these adverse life events (ALEs) experienced by South Africans accord with the findings of Brugha and colleagues [5]. They reported that a substantial amount of adversity measured in life was due to a relatively small group of life event categories i.e. 12 event categories that they described under the ‘List of Threatening Experiences’. These event categories include serious illness, injury and death of loved ones, among other major adversities. Considering the numerous ALEs, including those already alluded to, facing many South Africans, there is a need to untangle and examine these ALEs in detail. It is important to evaluate the burden of ALEs in the local context because of the unique South African history of apartheid, the current context of poverty and socioeconomic inequalities, and the high burden of HIV infection.

Moreover, an individual’s ability to cope with stressors and their perception of the ALE experienced may likely differentially influence their exposure to and success in coping with the stressors. Two theoretical constructs that attempt to identify an individual’s coping skills are Antonovsky’s sense of coherence (SOC) or orientation to life questionnaire and Rotter’s locus of control (LOC) concept. SOC assesses a personality and related ‘stress-resistance resource’ that comprises the belief that life is comprehensible, manageable and meaningful, and has been suggested to be important in coping with stressors and for maintenance of health [6]. LOC refers to the belief that individuals have in the amount of control they exert over their lives [7, 8]. Therefore, it may be hypothesised that individuals with better coping skills may experience fewer ALEs or vice versa.

To our knowledge, few studies have attempted to describe the burden of ALEs, the ability to cope with adversity and the impact of risky lifestyle behaviours and the effect of HIV-related ill-health in family members on experiences of ALEs. Therefore, this study aimed to describe the distribution of 12 ALEs by age and gender, and to examine the socio-demographic characteristics, risky lifestyle behaviours, psychosocial coping mechanisms and burden of HIV-related ill-health in family members that are associated with ALEs in 25-74-year-old black residents of Cape Town.

## Materials and methods

### Study population and sampling procedure

In 2008/09, a sample of predominantly black 25-74-year-old residents of five townships in Cape Town participated in this cross-sectional study. These data are from the Cardiovascular Risk in Black South Africans (CRIBSA) study that determined the prevalence of cardiovascular disease risk factors including obesity and physical activity levels in these townships. Considering that ill-health and other conditions are likely to impact on obesity and activity levels, individuals with certain ailments were excluded from this study. Individuals who were unable to give consent, on tuberculosis treatment, on antiretroviral therapy, bedridden, pregnant or lactating, resident in Cape Town for less than three months or had received cancer treatment within the last year were excluded. The sampling procedure, described in detail previously [9], included a 3-stage cluster sampling technique stratified by area and housing type. Quotas, calculated using the 2001 census, were pre-specified by age and gender categories.

### Data collection and definitions

Trained fieldworkers administered questionnaires pertaining to socio-demographic data (including education level, employment status, housing type and household assets), tobacco (WHO STEP-wise surveillance questionnaire) [10] and alcohol use, ALEs and psychosocial coping skills. The assets used to define wealth included ownership of consumer items (durable goods such as a radio, television, telephone/cellular phone, refrigerator and washing machine), dwelling characteristics such as wall and flooring materials, access to electricity, the source of drinking water and toilet facilities.

Current smokers were defined as those who smoked ≥1 cigarette a day or occasionally. The CAGE set of four yes/no questions assessed problematic alcohol use [11]. Problem drinking was deemed present if ≥2 of the CAGE questions were answered affirmatively. Having spent more than half their lives (≥50%) in urban areas defined urbanisation.

The Brugha ALEs questionnaire comprised 12 questions that related to negative life events such as illness, death, financial or marital difficulties, etc. and their impact [5]. A score of one was assigned for each category of ALE experienced. Their impact was categorised and scored as no impact (0), some impact (1) or significant impact (2), separately for an adverse event that occurred within the previous six months or more than six months ago.

The SOC and LOC scales assessed coping skills. Antonovsky’s 13-item SOC scale (S1 Table), previously validated in South Africa [12], measured comprehensibility (cognitive), manageability (instrumental/behavioural) and meaningfulness (motivational)) [13]. A low SOC inferred a poor ability to cope with stressors or to adapt to adversity i.e. low ‘resilience’ [6, 14]. Rotter’s LOC set of six questions (S2 Table) determined the individual’s perceived sense of control over his/her environment and life. A low score construed poor perceived control and a high score good perceived control [15].

This study was conducted at a time when HIV infection was highly stigmatised in South Africa and an extremely sensitive issue to address. This made direct enquiry about HIV infection difficult; therefore, indirect questions pertaining to the burden and impact of HIV infection in family members or the household were administered. Proxy questions included inquiring about the ill-health or death of a young family member or having to take care of a child whose parents were ill or had died, among others.

### Statistical analysis

Data analyses, conducted using STATA 15.0, included descriptive statistics such as crude prevalence, calculated using the weights based on the sample design and adjusted for the realised sample. A principal component analysis of the pooled data, based on the assets that defined wealth, was used to develop an asset index [16]. The ALEs were categorised into tertiles with cut-points ≤4 ALEs, 5–6 ALEs and ≥7 ALEs to present the bivariate associations.

Multivariable linear regression analyses determined the independent associations of the socio-demographic variables, coping skills and lifestyle behaviours with ALEs in a main effects model. Thereafter, each variable pertaining to the burden of HIV-related ill-health were modelled independently to avoid multicollinearity.

The University of Cape Town’s Research and Ethics Committee approved the study. All participants signed informed consent.

## Results

The realised sample of 1099 participants included 392 men and 707 women (64% and 108% of the planned sample, respectively). The response rate was 86%, and 42% of the non-responders were men i.e. 79 of the 187 selected people who the study team were unsuccessful in contacting.

The mean lifetime score of the 12 ALE categories experienced was 6.1 ±2.1 (range 0–12) with men reporting a significantly higher number of events compared with women (p<0.001) (Table 1). The impact of events experienced, however, was significantly higher in women for events occurring within the previous six months (p = 0.018) but significantly higher in men for events occurring >6 months ago (p<0.001).

**Table 1:** Prevalence (%) and means (±SD) of adverse life events in 25-74-year-old black adults presented by gender and age categories.

		Gender			Age categories in years					
	Total	Men	Women	p-value	25–34	35–44	45–54	55–64	65–74	p-value
Number	1099	392	707		357	261	254	150	77	
**Mean ±SD**										
Total lifetime score of adverse life event categories experienced	6.1 ±2.1	6.6 ±2.2	5.8 ±2.0	**<0.001**	5.6 ±1.9	6.3 ±2.2	6.4 ±2.3	6.3 ±2.0	5.6 ±1.8	**0.010**
Impact: events occurred ≤6 months ago	3.9±2.9	3.7±2.8	4.1±3.0	**0.018**	4.2±2.8	4.3±3.0	4.1±3.2	3.1±2.6	2.3±2.0	**<0.001**
Impact: events occurred >6 months ago	9.9±4.3	10.6±4.6	9.6±4.2	**<0.001**	8.6±3.9	10.5±4.5	10.8±4.7	10.9±3.9	9.5±3.5	**<0.001**
**Prevalence, %**										
You yourself suffered a serious illness, injury or an assault	48.2	57.8	39.6	**<0.001**	41.1	46.5	58.0	61.0	54.5	**<0.001**
A serious illness, injury or assault happened to a close relative of yours	53.5	51.2	55.6	0.251	48.3	56.7	58.8	55.7	57.2	**0.015**
Your parent, child or spouse died	74.2	73.1	75.3	0.485	58.5	75.5	90.2	97.1	96.8	**<0.001**
A close family friend or another relative (aunt, cousin, Grandparent) died	88.5	86.4	90.5	0.600	86.8	89.0	88.4	91.4	96.8	0.192
You had a separation caused by marital difficulties	19.0	20.0	18.1	0.473	6.9	21.6	33.8	35.0	20.8	**<0.001**
You broke off a steady relationship	58.6	62.6	55.0	**0.045**	61.4	62.8	55.8	52.4	39.3	**0.031**
You had a serious problem with a close friend, neighbour or relative of yours	36.8	34.4	39.1	0.167	35.6	39.7	36.6	39.7	31.7	0.678
You became unemployed or you were seeking work unsuccessfully for more than one month	72.3	75.9	68.9	0.058	80.7	78.5	69.2	42.9	34.0	**<0.001**
You were fired from your job	17.2	26.3	9.1	**<0.001**	16.4	21.5	16.7	15.5	10.7	0.323
You had a major financial crisis	81.2	81.5	80.9	0.820	79.9	82.4	86.0	83.1	71.1	0.076
You had problems with the police and a court appearance	26.0	37.9	15.3	**<0.001**	22.1	33.2	29.5	23.1	17.4	**0.046**
Something you valued was lost or stolen	39.9	50.0	30.8	**<0.001**	39.1	43.4	38.9	38.1	38.0	0.785

Mean ±SD are reported for the study sample and not adjusted for the population; significant p-values are in bold.

The most frequent ALE was the death of a close family friend or relative (88.5%) followed by a major financial crisis (81.2%) with no trend across gender or age group. Men, compared with women, were significantly more likely to have suffered a serious illness, injury or an assault (57.8% vs. 39.6%), broken off a steady relationship (62.6% vs. 55%), been fired from a job (26.3% vs. 9.1%), experienced problems with the police and a court appearance (37.9% vs. 15.3%) and had something valuable stolen or lost (50.0% vs. 30.8%).

Participants aged 35–64 years experienced significantly more ALEs compared with their younger or older counterparts (p = 0.010). Older participants compared with 25-34-year-olds were more likely to have suffered a serious illness, injury or an assault or have this happen to a close relative and to have experienced the death of a parent, child or spouse. However, 25-44-year-olds (78.5–80.7%) were most likely to have become unemployed or were seeking work unsuccessfully for more than one month. Having problems with the police and a court appearance was reported by a third of 35-44-year-olds followed by 45-54-year-olds (29.5%).

Participants who experienced ≥7 ALE categories (highest tertile) compared with ≤4 ALE categories (lowest tertile) were more likely to be male (55.6% vs. 36.1%) and to have spent >50% of their lives in urban areas (65.5% vs. 53.7%) (Table 2). They also had significantly lower SOC (50.8 vs. 59.0) and LOC (18.0 vs. 19.5) scores. Their lifestyles were unhealthier with significantly higher rates of problem drinking (42.9% vs. 20.5%) and daily smoking (31.4% vs. 19.1%). Family and personal burden of ill-health was significantly higher (p<0.001) with rising ALE tertiles except for HIV/AIDS in the household (p = 0.102).

**Table 2:** Socio-demographic characteristics, psychosocial coping mechanisms, lifestyle behaviours and burden of ill-health presented by adverse life event tertiles.

	≤4 life events	5–6 life events	≥7 life events	p-value
Number	258	392	449	
***Socio-demographic characteristics***				
Age in years, mean ±SD	42.7±13.5	43.0±13.3	44.0±11.9	0.167
Gender: male, %	36.1	44.7	55.6	**<0.001**
Education: ≤7 years of schooling, %	38.4	31.5	34.1	0.321
>50% of life spent in urban area, %	53.7	54.9	65.5	**0.003**
Employment Status, %:				0.980
Employed	23.5	22.7	22.6	
Unemployed	59.7	60.7	61.9	
Other*	16.9	16.6	15.4	
Housing type: informal shack, %	54.2	44.5	49.2	0.102
Poorest wealth tertile, %	35.4	38.8	36.2	0.736
***Coping skills*, *mean ±SD***				
Sense of coherence	59.0±9.7	55.8±9.8	50.8±10.0	**<0.001**
Locus of control	19.5±2.9	19.0±3.0	18.0±2.9	**<0.001**
***Unhealthy lifestyle behaviours*, *%***				
Drink alcohol	27.1	43.9	52.6	**0.002**
Problematic alcohol use: CAGE ≥2	20.5	29.1	42.9	**<0.001**
Currently smoke	25.2	31.2	37.4	**0.024**
Smoke: ≥1cigarette/day	19.1	28.2	31.4	**0.027**
***Family and personal burden of ill-health*, *%***				
Ill-health or death of a young family member	24.8	29.2	45.4	**<0.001**
Take care of a child whose parents are ill or have died	9.8	12.2	21.1	**<0.001**
Taking care of a child whose parents are ill or have died makes taking care of your own health difficult	0.5	2.5	7.0	**<0.001**
HIV/AIDS in the household	16.5	19.9	24.0	0.102
Own health perceived as being poor	15.0	16.6	24.3	**0.004**

Mean ±SD are reported for the study sample and not adjusted for the population; significant p-values are in bold; *Other: comprised pensioners, homemakers, students and those receiving disability grants.

In the multivariable linear regression model, ages 35–44 years, 45–54 years and 55–64 years but not 65–74 years, compared with 25-34-year-olds, were significantly associated with increasing number of ALEs (Table 3). Male gender, having spent >50% of life in urban areas, >7 years of education and unemployment were also significantly related to a greater number of ALEs. Problematic alcohol use but not daily smoking was linked to more ALEs. Determinants of psychosocial coping mechanisms i.e. SOC and LOC were significantly lower with increasing number of ALEs.

**Table 3:** Multivariable linear regression models for the associations with increasing number of adverse life events.

	Coefficient	95% Confidence Interval		p-value
		Lower limit	Upper limit	
Age categories in years: 25–34				
35–44	0.608	0.292	0.923	**<0.001**
45–54	0.772	0.412	1.132	**<0.001**
55–64	0.647	0.181	1.113	**0.007**
65–74	0.068	-0.453	0.590	0.796
Gender: male	0.726	0.467	0.985	**<0.001**
>50% of life spent in urban area	0.286	0.044	0.527	**0.021**
Education: >7 years of schooling	0.351	0.026	0.677	**0.034**
Housing type: informal shack	-0.169	-0.469	0.131	0.267
Poorest wealth tertile	-0.058	-0.367	0.251	0.712
Smoke: ≥1 cigarette/day	-0.058	-0.367	0.251	0.712
Problematic alcohol use: CAGE ≥2	0.564	0.265	0.862	**<0.001**
Increasing sense of coherence	-0.059	-0.071	-0.046	**<0.001**
Increasing locus of control	-0.079	-0.126	-0.032	**0.001**
Ill-health or death of a young family member	0.659	0.400	0.919	**<0.001**
Take care of a child whose parents are ill or have died	0.456	0.082	0,830	**0.017**
Taking care of a child whose parents are ill or have died makes taking care of your own health difficult	0.901	0.200	1.602	**0.012**
HIV/AIDS in the household	0.382	0.122	0.642	**0.004**
Own health perceived as being poor	0.236	-0.091	0.563	0.157

Significant p-values are in bold; When variables relating to family and personal burden of ill-health were entered individually and separately in the above model, there was no change in the direction or significance of the other variables in the models.

When the variables related to burden of ill-health were individually and separately entered in the above model, four of the five variables were significantly associated with higher numbers of ALEs. There was no change in the direction or significance of the other variables in any of these models.

## Discussion

This is among the few studies, to our knowledge, to describe the distribution of and associations with ALEs in a black urban community-based South African population. The mean number of ALEs categories experienced was high with death, financial crises and unemployment the most prevalent ALEs. These frequently experienced ALE categories were similar to those reported in a South African national mental health survey [17] and in HIV-infected individuals in Pretoria [18]. This is in keeping with the local socio-political and economic situation in South Africa, which has the highest burden of HIV infection globally, a high unemployment rate, widespread poverty and high socioeconomic inequity [2].

HIV/AIDS is the primary cause of mortality in the country contributing to a high burden in the working-age population [2, 19]. Albeit indirectly, this was reaffirmed in this study which was conducted prior to the widespread introduction of antiretroviral drugs for the treatment of HIV infection in South Africa; over a third (35.2%) of participants reported ‘ill-health or death of a young family member’ and one in five (21%) participants reported ‘HIV/AIDS in the household’. This was likely closely linked to 15% of participants having to ‘take care of a child whose parents are ill or have died’. Globally, about 17 million children have lost one or both parents to HIV/AIDS; 90% of whom reside in Sub-Saharan Africa [20]. This underscores that HIV/AIDS has a wide impact, not only on individuals with the infection but on those close to them. There are changes in the roles and structure of the family with greater responsibilities and financial strain placed on family members such as grandparents and the extended family [21]. The latter now have to fulfil the tasks of their deceased or ill relatives as caregivers and breadwinners; they may struggle to do so because of financial constraints or their own ill-health. This likely further exacerbates the cycle of poverty among the poor as breadwinners succumb to HIV/AIDS.

‘Conditioning variables’ including individual factors such as coping behaviours and social factors such as access to material resources or social support may modify the relationship among stressors, responses and health outcomes [22]. Two scores that evaluated coping behaviours in this study were SOC and LOC. The significant associations of lower SOC and LOC with increasing ALEs are indeed interesting. SOC assesses a personality and related ‘stress-resistance resource’ and has been suggested to be important in coping with stressors and for the maintenance of health [6]. A longitudinal study in Finland reported that ALE experiences lowered SOC in men and women [23]. However, findings of other studies suggest that the link between ALEs and SOC may perhaps be in the opposite direction. A longitudinal study of nurses in Sweden described fewer negative life events experienced by women with a high SOC vs. those with low SOC [24]. This was particularly true for controllable adverse events e.g. serious and long-lasting conflict with a close relative compared with uncontrollable adverse events e.g. death of a close friend/relative. This suggests that individuals with a lower SOC may be at greater risk to experience ALEs. However, causality cannot be established in this study because of the cross-sectional design. Further prospective research is required to determine causality between SOC and ALEs in the local setting. This is of interest because SOC has been suggested to play a mediating role between adversity and stressful experiences and psychological well-being [25]. The concept of LOC refers to the belief that individuals have in the amount of control they exert over their lives [8]. Individuals with low LOC who do not take responsibility for their actions may be prone to more ALEs. A longitudinal study in The Netherlands reported an inverse relationship between ALEs and LOC, similar to this study [26]. Further, greater ALEs predicted a lower LOC over time. There may perhaps be a bidirectional relationship between ALEs and LOC, which warrants further exploration.

Individuals with lower SOC or LOC may be prone to ‘stress proliferation’, a process where an initial stressor leads to additional stressors or ALEs [27]. Additionally, individuals with a sense of control or mastery over their lives may likely be buffered from the impacts of stressors and actively attempt problem-solving. Therefore, it may be important to address low SOC and LOC to enable individuals to not only cope better with ALEs but to perhaps even prevent some ALEs entirely. Further research is required for a better understanding of the link between stress exposure or ALEs and the lack of psychosocial coping skills i.e. the extent to which individuals’ stressful experiences and their coping resources play a role in exacerbating additional ALEs needs elaboration [27].

The high burden of ALEs and its relationship with lower SOC and LOC scores, manifestations of poor coping skills, has important implications for policymakers. Interventions that offer the opportunity to ease distress, promote problem-solving and encourage adaptation among individuals faced with major family, health, finance, work and environmental stressors are required. Coping skills as well as social support interventions that adequately cushion the effects of stress should be identified and incorporated into programmes implemented by community agencies, religious organisations, employers, schools, etc. [27].

The significant association of urbanisation with higher ALEs may reflect more adverse living circumstances and greater life stresses experienced in some urban compared with rural environments [28]. These may include violence, crime, alcohol misuse, overcrowding and housing shortages with poor sanitation, among other problems. With urbanisation occurring at a rapid rate in South Africa and globally, it is imperative for governments to pay greater and urgent attention to urban planning to ensure adequate living standards in urban centres.

That the markers of socioeconomic status such as housing type and wealth as determined by the asset index were not associated with ALEs is surprising; lower socioeconomic status is generally related to experiences of higher ALEs [22, 28]. However, this highlights the complex nature of the associations with protective factors such as social support possibly playing a modifying role in negating the effects of ALEs. Social relations and support may be protective during periods of stress because of emotional or practical assistance rendered [27, 29].

In contrast, the association of >7 years of education with ALEs was surprising but may possibly be related to an imbalance between giving and receiving social support [29]. For example, greater family expectations and responsibilities may place a higher burden on the more educated individual leading to increasing financial difficulties; 81% of participants experienced a major financial crisis. Examining dynamics such as household composition, social support and community characteristics may likely contribute to a greater understanding of these influences on experiences of ALEs [22, 29]. Notably, problem drinking was higher among more, compared with less, educated participants (36.5% vs. 28.2%, p = 0.029). Problem drinking may play a mediating role in the association of education with ALEs.

Participants who were problem drinkers were more likely to experience higher ALEs, which accords with the relation between stress and alcohol misuse described in the literature [30]. Similarly, a national US survey reported a consistent direct association between the number of stressors experienced in the previous year and all measures of heavy drinking [31]. Problem drinking is linked to many social problems including those assessed in this study; 42.9% of participants in the highest vs. 20.5% in the lowest ALEs tertile were problem drinkers. Alcohol misuse places a major burden on the health, economic and social well-being of the individual. On the other hand, unemployment and financial crises, which were highly prevalent in this study, may contribute to alcohol misuse. Unemployment is among the more stressful ALEs that adults may experience and frequently leads to financial strain, loss of self-esteem and decreased social status [32]. The relationship between unemployment and alcohol misuse is well documented in the literature [33] and accords with the findings of this study. A significantly higher proportion of participants who were unemployed compared with their counterparts were problem drinkers (38.3% vs. 26.1%, p = 0.008). Therefore, the inadequate focus on alcohol control in South Africa is disturbing and highlights the need for urgent action to address inequality, poverty and other social ills that are exacerbated by alcohol misuse.

### Limitations of this study

The low sample realisation in men (64%), which was due to difficulties encountered in making the initial contact with potential male participants was a limitation of this study. This is common in local epidemiological studies and necessitated higher sampling weights and a loss of precision. The cross-sectional study design precluded conclusions about causal associations between ALEs and the variables evaluated. Many factors including ALEs and tobacco and alcohol use were self-reported which may lead to same source bias. These study findings may be generalizable to other urban black South Africans residing in townships but not to rural settings because of differing experiences, lifestyle behaviours, and socioeconomic and environmental circumstances, etc.

## Conclusions

There was a high burden of ALEs, particularly death of loved ones, financial hardships and unemployment, experienced by urban black residents of Cape Town townships. ALEs were found to be linked to lower SOC and LOC, indicators of poor coping skills, and problem drinking, among other factors. Alcohol misuse, which exacerbates and perpetuates poverty, needs to be a priority target for government policy action. Furthermore, policymakers need to perhaps formulate and encourage programmes that improve coping mechanisms and promote problem-solving behaviours. Additional research exploring the link between stress exposure or ALEs and poor coping resources in exacerbating further stressors is required.

## Supporting information

S1 Table Sense of coherence scale (SOC-13). (DOCX)

S2 Table Locus of control scale. (DOCX)
