# Supplementary material for: A high burden of adverse life events and poor coping mechanisms experienced by urban-dwelling black South Africans
Source: PLoS One. 2020 Sep 10;15(9):e0238320. doi: 10.1371/journal.pone.0238320 (PMC7482925; doi:10.1371/journal.pone.0238320)
Supplement: S1 Table — (DOCX) [file pone.0238320.s001.docx]

**S1 Table: Sense of coherence scale (SOC-13)**

| *1) Do you have the feeling that you don’t really care about what goes on around you?* Very seldom or never [1]; Very often [7]* |
| --- |
| *2) Has it happened in the past that that you were surprised by the behaviour of people whom you thought you knew well?* Never happened [1]; Always happened [7]* |
| *3) Has it happened that people whom you relied/depended on disappointed you?* Never happened [1]; Always happened [7]* |
| *4) Until now your life has had:* No clear direction or purpose at all [1]; Very clear direction and purpose [7] |
| *5) Do you have the feeling that you’re being treated unfairly?* Very often [1]; Very seldom or never [7] |
| *6) Do you have the feeling that you are in an unfamiliar situation and don’t know what to do?* Very often [1]; Very seldom or never [7] |
| *7) Doing the things you do every day is*: A source of deep pleasure and satisfaction [1]; A source of frustration and boredom [7]* |
| *8) Do you feel confused or have very mixed-up feelings and ideas?* Very often [1]; Very seldom or never [7] |
| *9) Does it happen that you have feelings inside that you don’t like or would rather not feel?* Very often [1]; Very seldom or never [7] |
| *10) Many people—even those who are confident and successful—sometimes feel like losers in certain situations. How often have you felt this way in the past?* Never [1]; Very often [7]* |
| *11) When something happened, have you generally found that:* You over-estimated or underestimated its importance? [1]; You saw things in the right perspective? [7] |
| *12) How often do you have the feeling that there's little meaning in the things you do in your daily life?* Very often [1]; Very seldom or never [7] |
| *13) Sometimes people have strong feelings that they cannot keep under control. How often do you have feelings that you're not sure you can keep under control?* Very often [1]; Very seldom or never [7] |

SOC scale is based on a Likert scale of 1-7. *Scoring for items 1, 2, 3,7 and 10 were reversed when total scores were calculated
