# Supplementary material for: A high burden of adverse life events and poor coping mechanisms experienced by urban-dwelling black South Africans
Source: PLoS One. 2020 Sep 10;15(9):e0238320. doi: 10.1371/journal.pone.0238320 (PMC7482925; doi:10.1371/journal.pone.0238320)
Supplement: S2 Table — (DOCX) [file pone.0238320.s002.docx]

**S2 Table: Locus of control scale**

| 1) In your general work, you feel you have control over what happens in most situations |
| --- |
| 2) You feel what happens in your life is often determined by factors beyond your control* |
| 3) Over the next 5-10 years, you expect to have more positive than negative experiences |
| 4) You often have the feeling you are being treated unfairly* |
| 5) In the past 10 years your life has been full of changes without you knowing what will happen next* |
| 6) You gave up trying to better your life a long time ago* |

Scoring: Strongly disagree [1]; Disagree [2]; Neutral [3]; Agree [4]; Strongly agree [5]. *Scores for items 2, 4, 5 and 6 were reversed when total scores were calculated
